# Torque teno virus for risk stratification of graft rejection and infection in kidney transplant recipients—A prospective observational trial

**DOI:** 10.1111/ajt.15810

**Published:** 2020-03-08

**Authors:** Konstantin Doberer, Martin Schiemann, Robert Strassl, Frederik Haupenthal, Florentina Dermuth, Irene Görzer, Farsad Eskandary, Roman Reindl‐Schwaighofer, Željko Kikić, Elisabeth Puchhammer‐Stöckl, Georg A. Böhmig, Gregor Bond

**Affiliations:** ^1^ Division of Nephrology and Dialysis Department of Medicine III Medical University Vienna Vienna Austria; ^2^ Division of Virology Department of Laboratory Medicine Medical University Vienna Vienna Austria; ^3^ Center for Virology Medical University Vienna Vienna Austria

**Keywords:** biomarker, complication: infectious, immunosuppression/immune modulation, infection and infectious agents—viral, infectious disease, kidney transplantation/nephrology, monitoring: immune, rejection, translational research/science

## Abstract

The nonpathogenic and ubiquitous torque teno virus (TTV) is associated with immunosuppression in solid organ transplant recipients. Studies in kidney transplant patients proposed TTV quantification for risk stratification of graft rejection and infection. In this prospective trial (DRKS00012335) 386 consecutive kidney transplant recipients were subjected to longitudinal per‐protocol monitoring of plasma TTV load by polymerase chain reaction for 12 months posttransplant. TTV load peaked at the end of month 3 posttransplant and reached steady state thereafter. TTV load after the end of month 3 was analyzed in the context of subsequent rejection diagnosed by indication biopsy and infection within the first year posttransplant, respectively. Each log increase in TTV load decreased the odds for rejection by 22% (odds ratio [OR] 0.78, 95% confidence interval [CI] 0.62‐0.97; *P *= .027) and increased the odds for infection by 11% (OR 1.11, 95% CI 1.06‐1.15; *P *< .001). TTV was quantified at a median of 14 days before rejection was diagnosed and 27 days before onset of infection, respectively. We defined a TTV load between 1 × 10^6^ and 1 × 10^8^ copies/mL as optimal range to minimize the risk for rejection and infection. These data support the initiation of an interventional trial assessing the efficacy of TTV‐guided immunosuppression to reduce infection and graft rejection in kidney transplant recipients.

## Graphical abstract

This trial defines an optimal plasma level range of the nonpathogenic and ubiquitous torque teno virus, which reflects the immunosuppression of its host, to minimize the risk for rejection and infection in kidney allograft recipients in the first year after transplantation. Fernández‐Ruiz comments on page 1963.

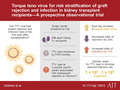

**Abbreviations**
ABMR: antibody‐mediated rejection
BL: borderline
c/mL: copies/milliliter
CI: confidence interval
DSA: donor‐specific antibodies
IQR: interquartile range
NPV: negative predictive value
OR: odds ratio
PPV: positive predictive value
pPVN: presumptive polyomavirus nephropathy
PVN: polyomavirus nephropathy
TCMR: T cell–mediated rejection
TTV: torque teno virus

## 1 INTRODUCTION

Kidney transplantation represents the only curative treatment for patients with end‐stage renal disease. After transplantation, immunosuppressive drugs are crucial to reducing the risk of organ rejection. Apart from this desired effect, immunosuppression increases the risk for infectious disease, being one of the leading causes of death after kidney transplantation.^1^ Moreover, current immunosuppression regimens are unable to sufficiently control allorecognition, which is reflected in chronic graft rejection being the leading cause of organ dysfunction.^2^ Therefore, the optimal management of immunosuppressive drug dosing requires a delicate balance between the risk of graft rejection due to inadequate immunosuppression and the deleterious side effects of excessive immunosuppression. At present, no diagnostic test or algorithm exists for guidance of immunosuppression in clinical routine.^3^ Currently, immunologic monitoring relies primarily on the quantification of calcineurin inhibitor drug trough level, which correlates more closely with the risk of drug‐related toxicity than immunosuppressive efficacy.^4^ Thus, there is urgent need for tools capable of personalizing immunosuppressive medication in order to simultaneously reduce the risk of infectious disease and graft rejection following kidney transplantation.

Two assays to characterize the immune function of solid organ recipients were proposed,^5^, ^6^ but none of them reached clinical practice to date. A test of leukocyte function, the QuantiFERON Monitor (Qiagen, Germany), was prognostic for infectious events in kidney, liver and lung transplant patients.^5^ In a randomized controlled trial, tailoring immunosuppression after liver transplantation via the assessment of CD4^+^ lymphocyte function, using ImmuKnow® (Cylex, Germany), resulted in less infectious events.^6^ However, the ideal candidate for the guidance of immunosuppression would reduce both graft rejection and infectious disease. Monitoring of the peripheral blood copy numbers of torque teno virus (TTV) appears to be a promising new strategy for characterization of the immune system of solid organ recipients.^7^ TTV is considered a surrogate marker of T cell function, but recent data proposed that TTV might also mirror humoral and innate immunity.^8^, ^9^, ^10^, ^11^, ^12^, ^13^ TTV was not proven to cause any human disease,^7^ the prevalence in solid organ recipients is up to 99%,^14^ and the virus is unaffected by conventional antiviral drug therapy.^15^ Peripheral blood TTV copy numbers might mirror the intensity of host immunosuppression.^16^ TTV load was shown to associate with the amount and type of immunosuppressive drugs administered to solid organ recipients and is thus indirectly associated with allograft rejection and infectious disease.^8^, ^15^, ^17^, ^18^, ^19^, ^20^, ^21^, ^22^, ^23^, ^24^, ^25^, ^26^, ^27^

In kidney transplant recipients, recent data suggest a potential of TTV quantification for risk prediction of infectious events^23^, ^25^ and graft rejection.^24^, ^27^ However, no prospective trial analyzed the association of TTV and rejection beyond month 1 after transplantation and data concerning TTV load and nonopportunistic infections are scarce. This large prospective observational trial was designed to assess the potential of TTV load for risk prediction of both infection and rejection in the first year after transplantation. The specific objective of this trial was to provide TTV cutoff level defining an optimal TTV range as a basis for a randomized controlled interventional trial to test the efficacy of TTV‐guided immunosuppression.

## 2 MATERIALS AND METHODS

### 2.1 Patient cohort and study design

The prospective observational “TTV Quantification for the Prediction of Organ Rejection in Kidney Transplantation; TTV‐POET” trial included all 386 consecutive adult (≥18 years of age) recipients of a kidney allograft transplanted at the Medical University Vienna, Austria, between January 1, 2016 and June 30, 2018. Patients were followed at the outpatient clinic of the Medical University Vienna for 12 months after transplantation or until dropout due to change of outpatient care center, graft loss, or death. The present study was approved by the local institutional review board (approval number: 1785/2016) and registered at the German Clinical Trials Registry (register number: DRKS00012335). An interim analysis of the present study including patients transplanted in 2016, restricted to infectious events, has been published elsewhere.^23^

### 2.2 Primary and main secondary outcome

The primary outcome of the TTV‐POET trial was predefined as biopsy proven graft rejection according to Banff classification. Protocol biopsies were not included in this analysis. Immunohistochemical assessment of C4d staining was evaluated on paraffin‐embedded sections. Multilayering of basement membranes of peritubular capillaries and glomerular basement membranes were assessed by electron microscopy. Histopathological lesions were categorized following the 2015 and 2017 updates of the Banff classification.^28^, ^29^ Infectious events were defined as any bacterial, fungal, or viral infection requiring antimicrobial or antiviral treatment, reduction of immunosuppressive drugs, hospitalization, or prolongation of a hospital stay. Polyomavirus infections were defined according to recommendations of the Banff working group and the American Society of Transplantation Infectious Diseases Community of Practice guidelines.^30^, ^31^ Plasma BK viral load ≥ 1 × 10^4^ copies/milliliter (c/mL) was defined as presumptive polyomavirus nephropathy (pPVN) and polyomavirus nephropathy (PVN) was defined by biopsy. Cytomegalovirus (CMV) syndrome was defined by CMV replication and the presence of attributable symptoms according to current consensus definitions.^32^ CMV viremia was not treated and thus not scored as infectious event. Fungal infections were proven cases of invasive fungal defined by the EORTC‐MSG consensus group.^33^ The following pathogens were classified as “opportunistic”: CMV, BK polyomavirus, *Aspergillus*, and *Pneumocystis jirovecii.*

### 2.3 Baseline immunosuppression and rejection therapy

All patients received triple immunosuppression with tacrolimus, mycophenolic acid, and corticosteroids. All HLA compatible patients received induction therapy with interleukin‐2 receptor blockade (Simulect; Novartis AG, Switzerland). Recipient of an HLA‐incompatible kidney, defined by preformed donor‐specific anti‐HLA antibodies (DSA), underwent IgG immunoapheresis and depleting antibodies (Gravalon; Neovii, Switzerland) according to a local protocol.^34^ Recipients of an ABO‐incompatible kidney underwent ABO blood group antigen–specific immunoapheresis (Glycosorb; Glycorex Transplantation AB, Sweden) and, in cases of AB antibody titers > 1:256, received additional induction therapy with CD20 antibody (Rituximab; Hoffmann‐La Roche, Switzerland) 4 weeks before transplantation. T cell–mediated rejection (TCMR) type I and II and borderline (BL) changes suspicious for TCMR were treated with 100 mg dexamethasone for 3 days and TCMR rejection type III or steroid refractory TCMR with 3 mg/kg depleting antibodies (Gravalon) for 10 days. Antibody‐mediated rejection (ABMR) was treated with immunoadsorption therapy^35^ and in case of refractory rejection with additional membrane filtration.^36^

### 2.4 Infection prophylaxis and monitoring

All patients received prophylaxis with trimethoprim‐sulfamethoxazole for 6 months after transplantation with 50% of the therapeutic dose adapted to kidney function and valganciclovir both for 3 months in CMV IgG‐negative recipients of a CMV IgG‐positive organ, in case of Epstein Barr virus IgG‐negativity and after treatment with depleting antibodies and IgG immunoapheresis with 50% of the therapeutic dose adapted to kidney function. Screening for CMV and BK polyomavirus after transplantation was performed by polymerase chain reaction (PCR) from peripheral blood once per week until discharge from the ward, on the first visit at the outpatient clinic, on month 3 after transplantation, and every 3 months thereafter. Epstein‐Barr virus PCR from peripheral blood was performed in Epstein‐Barr virus IgG‐negative recipients on the first visit at the outpatient clinic, 1 month after transplantation, monthly until month 6 after transplantation, and every 3 months thereafter. We did not perform specific screening for other viral infections or bacterial and fungal infections.

### 2.5 TTV quantification

TTV was quantified prospectively per protocol in the peripheral blood at the following predefined time points: before transplantation and after transplantation once per week until discharge from the ward, on the first visit at the outpatient clinic, on month 3 after transplantation, and every 3 months thereafter. Treating physicians were unaware of the TTV results. TTV DNA was extracted from 200 μL of plasma using the NucliSENS easyMAG platform (bioMeriéux, France), as recommended by the manufacturer. TTV DNA was quantitated by TaqMan real‐time PCR, as described previously with laboratory developed primers.^8^, ^37^ The quantitative PCR reactions were performed in a volume of 25 µL using 2 × TaqMan Universal PCR Master Mix, containing 5 µL of extracted DNA, 400 nmol/L of each primer, and 80 nmol/L of the probe. Thermal cycling was started for 3 minutes at 50°C, followed by 10 minutes at 95°C, and then by 45 cycles at 95°C for 15 seconds, at 55°C for 30 seconds, and at 72°C for 30 seconds, using the CFX96 Real‐time System (Bio‐Rad, Hercules, CA). Linear range was from 1 × 10^3^ to 1 × 10^11^ c/mL and limit of detection was 7 × 10^2^ c/mL.

### 2.6 Statistical analysis

The Mann‐Whitney *U* test was used to compare TTV between groups. Generalized linear models were used to estimate the effect size of the association between allograft rejection and infection, respectively and TTV. Effect size was displayed as odds ratio (OR) and 95% confidence interval (95% CI). For sensitivity analysis restricted to a single event per person, the first event after transplantation was selected. Potential confounders of the effect size of the association between TTV and rejection and infection, respectively, were assessed using bivariate analysis. Potential effect modifiers were tested using Mantel‐Haenszel strata. A change in the effect size of > 10% was defined as significant. For multivariate analysis, co‐variables were selected on the basis of clinical relevance. For multivariable modeling, backward elimination was used and the “rule of 10” was applied to define the maximum of variables. A *P* < .05 was the predefined limit of significance. Log normally distributed variables were log transformed. MS EXCEL 2010 (Microsoft), IBM SPSS Statistics 24.0 (SPSS Inc), and STATA 15 (Stata Corp., College Station, TX) were applied for data analysis.

## 3 RESULTS

### 3.1 Baseline and outcome data of the total study cohort

A total of 386 patients received a kidney transplant at the Medical University Vienna between January 1, 2016 and June 30, 2018. Median recipient age at transplantation was 55 years, 35% were female, 19% had a history of prior kidney transplantation, 82% received a kidney from a deceased donor, and 9% had preformed DSA (Table 1). One‐year patient survival was 95.6% and one‐year death censored graft survival was 94.8%. Causes of death and graft loss are displayed in Table S1. Median follow‐up time was 296 days. Graft rejection was diagnosed in 68 of the recipients (18%) within the first year after transplantation and a total 71 episodes of rejection were documented. Rejections scored according to Banff classification are shown in Table S2. A total of 207 recipients (54%) had 472 episodes of infection within the first year after transplantation. Details on affected organ systems and causative pathogens are displayed in Table 2.

**Table 1:** Baseline characteristics of the total study cohort and the cohort selected for analysis of the association between TTV load and rejection and infection, respectively

	Total cohort (n = 386)		Biopsy cohort (n = 37)		Infection cohort (n = 274)
Recipient characteristics					
Age; years, median (IQR)	55 (44‐64)	54 (44‐62)		54 (44‐63)	
Female sex	135 (35)	12 (32)		91 (33)	
Cardiovascular disease^a^	76 (20)	7 (19)		57 (21)	
Cause of end‐stage renal disease					
Immunologic	84 (22)	9 (24)		56 (20)	
Cystic kidney disease	69 (18)	6 (16)		52 (19)	
Diabetes	50 (13)	4 (10)		34 (12)	
Hypertension	39 (10)	4 (10)		24 (9)	
Hereditary	30 (8)	3 (8)		33 (12)	
Other	48 (12)	4 (10)		29 (11)	
Undefined cause	66 (17)	7 (19)		46 (17)	
Time on dialysis; years, median (IQR)	2.6 (1.3‐4.4)	2.6 (1.1‐4.9)		2.6 (1.3‐4.1)	
Donor characteristics					
Deceased donor	318 (82)	30 (81)		230 (84)	
Donation after circulatory death	34 (9)	2 (5)		22 (8)	
Donor age; years, median (IQR)	55 (44‐67)	56 (47‐67)		55 (45‐66)	
Donor female	194 (50)	25 (68)		123 (45)	
Transplant characteristics					
Retransplantation	74 (19)	9 (24)		46 (17)	
ABO‐incompatible transplantation	25 (6)	2 (5)		17 (6)	
HLA‐A/B/DR mismatch; N, median (IQR)	3 (2‐4)	3 (2‐4)		3 (2‐4)	
Donor‐specific antibody	34 (9)	7 (19)		20 (7)	
Cold ischemia time; hours, median (IQR)	13 (7‐17)	15 (7‐18)		13 (8‐18)	
Delayed graft function^b^	132 (32)	12 (32)		81 (30)	
CMV donor IgG^+^/recipient IgG^‐^	52 (14)	5 (14)		39 (14)	
CMV prophylaxis	110 (28)	11 (30)		75 (27)	

Note

Data are presented as number (%) unless otherwise indicated.

Abbreviations: CMV, cytomegalovirus; HLA, human leukocyte antigen; IgG, Immunoglobulin G; IQR, interquartile range; N, number, TTV, torque teno virus.

a History of myocardial infarction, coronary angioplasty, and/or stent or coronary surgery.

b Delayed graft function was defined by the necessity of >1 renal replacement therapy posttransplant.

**Table 2:** Affected organ system and causative pathogen of infectious events in the total cohort and the cohort selected for analysis of the association between TTV load and infection

	Total cohort (n = 386)	Infection cohort (n = 274)	
Organ system			
Urinary tract	219 (46)		80 (49)
Respiratory system	60 (13)		34 (17)
PVAN/pPVAN	37 (8)		21 (11)
Gastrointestinal	36 (8)		15 (8)
Skin and soft tissue	26 (6)		7 (4)
Other	15		8
Bacteriemia	78		35
Bacteria			
*Escherichia coli*	106 (40)		40 (43)
*Enterococcus* species	54 (21)		16 (17)
*Klebsiella* species	27 (10)		12 (13)
*Pseudomonas* species	21 (8)		7 (7)
*Staphylococcus* species	15 (6)		5 (5)
*Clostridium* species	10 (4)		3 (3)
Other	29		11
Virus			
CMV^a^	58 (50)		32 (54)
BKV^b^	37 (32)		21 (36)
Influenza A/B	9 (8)		3 (5)
Other	13		3
Fungi			
*Candida* species^c^	10 (67)		2 (40)
*Pneumocystis* *jiroveci* d	4 (27)		3 (60)
*Aspergillus fumigatus* e	1 (7)		0 (0)

Note

Data are presented as number (%). Percentages are calculated from the total number of affected organ systems and bacterial, viral, and fungal infections, respectively.

Abbreviations: BKV, BK polyomavirus; CMV, cytomegalovirus; PVN, polyomavirus associated nephropathy; pPVN, presumptive polyomavirus nephropathy, TTV, torque teno virus.

a Seven episodes of colitis, 1 episode of pneumonia.

b Twenty episodes of PVN, 17 episodes of pPVN.

c Five episodes of soft tissue infection, 4 episodes of esophagitis, 1 episode of kidney graft infection.

d Four episodes of pneumonia.

e Pulmonary aspergillosis.

### 3.2 TTV quantification in the context of allograft rejection

**Figure 1:**
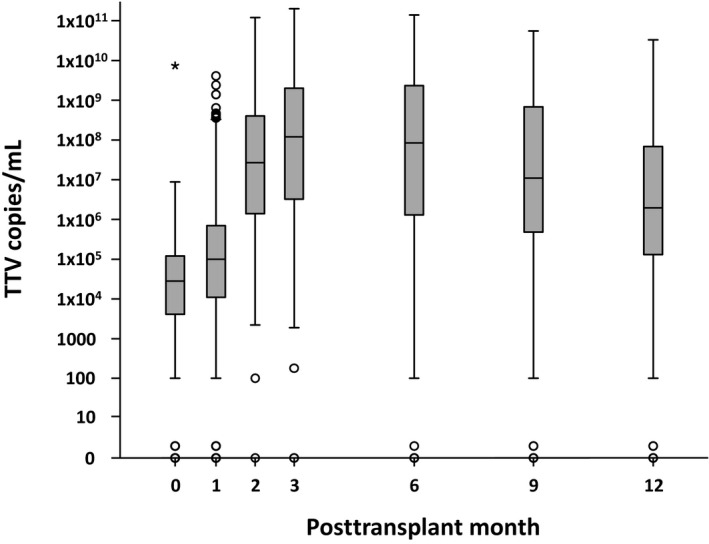
All torque teno virus (TTV) measurements (n = 3265) of the total cohort (n = 386) are included. On the y‐axis TTV copies per mL (c/mL) peripheral blood and on the x‐axis time since transplantation in months are plotted. TTV load is displayed in box plots combining all measurements closest to the month described on the x‐axis. The box represents the 25th and 75th percentile. The horizontal line in the box represents the median. The whiskers represent approximately 95% of the data, circles represent outliers, and asterisks represent extreme outliers (>3 times the height of the boxes). Median pretransplant TTV load was 10^4^ c/mL. After transplantation, TTV load quickly increased and reached a peak at month 3 (10^8^ c/mL). Thereafter TTV slowly decreased up to month 12 (10^6^ c/mL)

Plasma for TTV analysis was sampled before transplantation and longitudinally per‐protocol after transplantation in all 386 patients. In total 3265 TTV quantifications were performed. Prior to transplantation, 88% of the patients had detectable TTV in the peripheral blood (median 3 × 10^4^ c/mL, interquartile range [IQR] 4 × 10^3^‐2 × 10^5^ c/mL; Figure 1). Posttransplant TTV load increased and peaked at day 101 (median; IQR 85‐165 days; TTV median 8 × 10^8^ c/mL, IQR 4 × 10^7^‐5 × 10^9^ c/mL). All pretransplant TTV‐negative patients except two turned TTV positive after transplantation.

**Figure 2:**
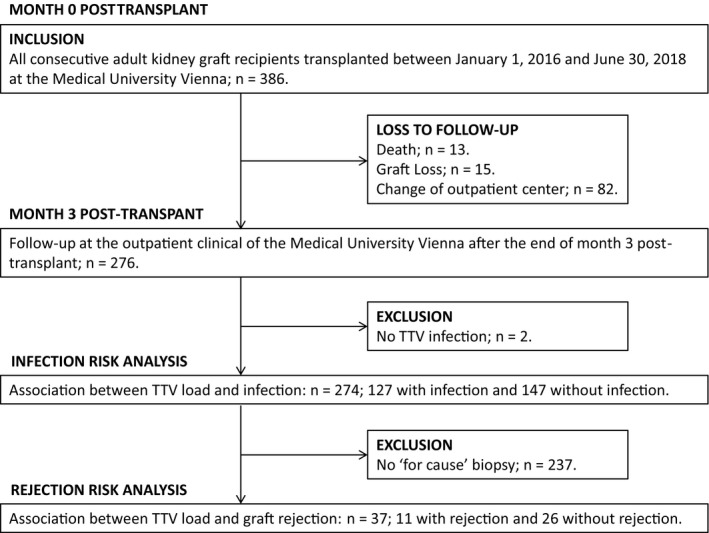
Between January 1, 2016 and June 30, 2018, 386 patients received a kidney graft at the Medical University Vienna. After month 3 posttransplant, 276 patients were still followed at the outpatient clinic of our center and 110 were lost to follow‐up due to death, graft loss, or change of center providing the outpatient care. Two patients were excluded because no torque teno virus (TTV) infection was detected. A total of 274 patients were included in the analysis of the association between infection and TTV level. A total of 37 patients had a “for cause” graft biopsy after the end of month 3 posttransplant and were included in the analysis of the association between graft rejection and TTV

In order to determine the potential of peripheral TTV load for risk prediction of graft rejection, all TTV measurements taken after TTV load stabilization at the end of posttransplant month 3 with an available subsequent “for cause” biopsy were analyzed. From the total cohort of 386 patients, 276 patients were still followed at our outpatient center after the end of month 3 posttransplant (Figure 2). Eighty‐two patients changed outpatient center, 15 lost their graft, and 13 died. Two patients without TTV infection were excluded. Applying these inclusion and exclusion criteria a total of 274 patients were available for further analysis. Of these 274 patients, 37 individuals had a graft biopsy with available preceding TTV quantification after month 3 posttransplant; 11 patients had rejection and 26 had no rejection. Two patients had repeated biopsies. Therefore, a total of 39 biopsies were available for the assessment of the association between TTV load and the risk of subsequent graft rejection.

Samples for TTV quantification were taken at a median of 154 days after transplantation (IQR 99‐258) and preceded subsequent biopsies by a median of 14 days (IQR 1‐15). Baseline characteristics of the 37 patients are detailed in Table 1. Of the 39 analyzed biopsies, 11 showed signs of allograft rejection. TCMR was detected in 5 (2 BL lesions TCMR, 3 TCMR type I), acute ABMR in 2, and mixed rejection in 4 biopsies (3 ABMR plus BL lesions, 1 ABMR plus TCMR type I; Table S2), and 28 biopsies showed no rejection.

Patients with allograft rejection had lower levels of TTV compared to patients without rejection (median 3.5 × 10^6^ c/mL, IQR 1.7 × 10^5^‐1.3 × 10^8^ c/mL vs median 2.5 × 10^8^ c/mL, IQR 5.8 × 10^6^‐9.3 × 10^8^ c/mL; *P *= .028) in subsequent biopsies. The odds for rejection decreased by 22% with every log level increase of TTV (OR 0.78, 95% CI 0.62‐0.97; *P *= .027). Sensitivity analysis restricted to one biopsy per patient (n = 37) and biopsies without BL lesions (n = 37), respectively, showed similar effect size of the association between TTV level and rejection, respectively (OR 0.80, 95% CI 0.64‐1.00; *P *= .05 and OR 0.76, 95% CI 0.60‐0.98; *P *= .032). Applying receiver operating curve, an area under the curve of 0.73 (IQR, 0.54‐0.92; *P *= .028) was calculated to classify rejection by TTV level (Figure S1A). A TTV level cutoff of 1.5 × 10^6^ c/mL corresponded to a specificity of 89% and a sensitivity of 36%, a negative predictive value (NPV) of 77%, and a positive predictive value (PPV) of 50%. Diagnostic accuracy for each log level of TTV is displayed in Table 3. For TTV levels above 10^7^ c/mL, high NPVs (range 84%‐87%) were calculated and PPV was high below TTV level of 10^5^ c/mL (range 57%‐85%).

**Table 3:** Diagnostic accuracy to detect rejection by TTV level

TTV^a^	NPV^b^	PPV^b^	Sensitivity^b^	Specificity^b^
10^4^	0.74‐0.76	0.76‐1.00	0.09‐0.18	1.00‐1.00
10^5^	0.77‐0.82	0.57‐0.85	0.18‐0.36	0.93‐0.96
10^6^	0.77‐0.85	0.47‐0.56	0.36‐0.64	0.75‐0.89
10^7^	0.84‐0.87	0.40‐0.50	0.64‐0.73	0.61‐0.71
10^8^	0.80‐0.89	0.30‐0.41	0.73‐0.91	0.18‐0.57
10^9^	0.67‐1.00	0.28‐0.30	0.91‐1.00	0.07‐0.14

Abbreviations: NPV, negative predictive value; PPV, positive predictive value; TTV, torque teno virus.

a Measurements have been grouped according to log level TTV copies/mL.

b Range.

To test whether TTV was independently associated with graft rejection, potential confounders were analyzed and adjusted ORs calculated (Table 4). None of the tested variables, including recipient age and sex, history of prior transplantation or preformed DSA, rejection episodes and posttransplant DSA prior to TTV assessment, graft function and immunosuppression at the day of TTV assessment, and timing of TTV assessment, changed the effect size of the association between TTV level and rejection.

**Table 4:** Unadjusted and adjusted effect size of the association between TTV and rejection

Method	Covariables	Odds ratio	95% Confidence interval
Unadjusted		0.78	0.62‐0.97
Adjusted^a^	Recipient age at transplantation	0.77	0.62‐0.97
	Recipient sex	0.79	0.64‐0.99
	History of prior transplantation	0.77	0.62‐0.97
	Preformed donor‐specific antibodies	0.78	0.62‐0.97
	Tacrolimus trough level at TTV assessment	0.78	0.62‐0.97
	Full dose mycophenolic acid at TTV assessment	0.78	0.62‐0.97
	Estimated glomerular filtration rate^b^ at TTV assessment	0.78	0.62‐0.98
	Posttransplant donor–specific antibodies	0.76	0.60‐0.96
	Graft rejection before TTV assessment	0.77	0.62‐0.97
	Time between kidney transplantation and TTV assessment	0.82	0.64‐1.06

Abbreviation: TTV, torque teno virus.

a Due to the limited event rate, no analysis on potential effect modifier and multivariable modeling was performed, respectively.

b Calculated by the Modification of Diet in Renal Disease equation.^38^

### 3.3 TTV quantification in the context of infectious disease

To determine the potential of TTV load for risk stratification of infection, we included all TTV measurements taken after month 3 posttransplant and analyzed if an infectious event was documented until the subsequent TTV assessment. All 276 patients, who were still followed at our outpatient clinic (Figure 2) after month 3 posttransplant, were included and two patients without TTV infection were excluded. Applying these criteria, a total of 274 patients with 785 TTV measurements were available for subsequent analysis. Baseline characteristics of the 274 included patients are detailed in Table 1. TTV measurements were followed by an infectious event 193 times (25%) and no infectious event was documented 592 times; 127 patients had one or more infections (46%) after month 3 posttransplant and 147 had no infectious event (54%). Median time of TTV assessment was 180 days (IQR 128‐252) after transplantation and median time from TTV quantification to infection was 27 days (IQR 12‐45 days). Most of the infections involved the urinary tract (49%; Table 2), followed by infections of the respiratory system (21%), infections of the allograft (13%), and infections of the gastrointestinal tract (9%). In 72% of the infections, a causative pathogen could be isolated and a total of 158 pathogens were described (Table 2). The most frequent pathogens were bacteria (59%), followed by virus (37%) and fungi (3%). Opportunistic pathogens were documented in 28% of the infections and hospitalization was required in 42%.

Quantification of TTV in the 785 events included in this analysis revealed higher level of TTV in the plasma drawn from individuals experiencing an infectious event in the subsequent observation period, compared to patients without infection (median 3.9 × 10^8^ c/mL, IQR 7.9 × 10^6^‐3.3 × 10^9^ c/mL vs median 2.6 × 10^7^ c/mL, IQR 1.3 × 10^6^‐9.2 × 10^8^ c/mL; *P *< .001). The odds for an infection increased by 11% with every log level increase of TTV (OR 1.11, 95% CI 1.06‐1.15; *P *= .001). Sensitivity analysis including only one event per patient (n = 274) showed similar effect size for the association between TTV level and infection (OR 1.10, 95% CI 1.02‐1.17; *P *= .01). Further subgroup analysis showed a comparable effect size for the association between TTV level and infections which did not require hospitalization (OR 1.10, 95% CI 1.04‐1.16, *P *< .001). The largest effect size was calculated for BK infections (pPVN and PVN; OR 1.21, 95% CI 1.06‐1.39, *P *= .005) followed by CMV disease (CMV syndrome and end‐organ disease; OR 1.16, 95% CI 1.05‐1.27, *P *= .005) and infections restricted to opportunistic pathogens (OR 1.16, 95% CI 1.07‐1.26, *P *< .001). A smaller effect size was found for infections with extracellular bacteria (OR 1.06, 95% CI 1.00‐1.12, *P *= .05). Applying receiver operating curve, an area under the curve of 0.62 (IQR, 0.58‐0.67; *P *< .001) was calculated to classify infection by TTV level (Figure S1B). A TTV level > 5.8 × 10^9^ c/mL corresponded to a specificity of 90%, a sensitivity of 18%, a NPV of 77% and a PPV of 37% to detect infection. Diagnostic accuracy for each log level of TTV is displayed in Table 5. For TTV level up to 10^9^ c/mL high NPVs were calculated for the detection of infection (range 100% to 79%) and the highest PPV was calculated for TTV loads above 10^10^ c/mL (range 44%‐67%).

**Table 5:** Diagnostic accuracy to detect infection by TTV level

TTV^a^	NPV^b^	PPV^b^	Sensitivity^b^	Specificity^b^
10^4^	0.83‐1.00	0.25‐0.25	0.93‐0.97	0.05‐0.10
10^5^	0.79‐0.83	0.25‐0.26	0.85‐0.92	0.10‐0.22
10^6^	0.82‐0.84	0.26‐0.29	0.74‐0.85	0.22‐0.42
10^7^	0.82‐0.84	0.29‐0.33	0.63‐0.73	0.43‐0.59
10^8^	0.80‐0.83	0.33‐0.36	0.41‐0.62	0.59‐0.76
10^9^	0.75‐0.80	0.36‐0.43	0.10‐0.41	0.77‐0.96
10^10^	0.75‐0.76	0.43‐0.67	0.01‐0.10	0.96‐0.99

Abbreviations: NPV, negative predictive value; PPV, positive predictive value; TTV, torque teno virus.

a Measurements have been grouped according to log level TTV copies/mL.

b Range.

To test whether TTV was independently associated with infection, potential confounders were analyzed and adjusted ORs calculated (Table 6). None of the tested variables including recipient age and sex, preformed DSA, ABO incompatible transplantation, CMV prophylaxis, rejection episodes before TTV assessment, posttransplant diabetes mellitus, graft function, leukocyte count and immunosuppression at the day of TTV assessment, and timing of TTV assessment changed the effect size of the association between TTV level and rejection. Recipient age and sex and posttransplant diabetes mellitus did not modify the effect size of the association between TTV level and infection.

**Table 6:** Unadjusted and adjusted effect size of the association between TTV and infection

Method	Covariables	Odds ratio	95% Confidence interval
Unadjusted		1.11	1.06‐1.15
Adjusted	Recipient age at transplantation	1.11	1.06‐1.15
	Recipient age at transplantation, >54 y	1.06	1.00‐1.12
	Recipient age at transplantation, ≤54 y	1.16	1.08‐1.15
	Recipient sex	1.10	1.06‐1.23
	Recipient sex, female	1.08	1.02‐1.14
	Recipient sex, male	1.13	1.06‐1.20
	CMV prophylaxis	1.11	1.06‐1.15
	ABO‐incompatible transplantation	1.11	1.06‐1.15
	Preformed DSA	1.11	1.06‐1.15
	Posttransplant diabetes mellitus	1.11	1.06‐1.15
	Posttransplant diabetes mellitus, yes	1.08	1.02‐1.16
	Posttransplant diabetes mellitus, no	1.12	1.06‐1.18
	Time between kidney transplantation and TTV assessment	1.10	1.05‐1.15
	Graft rejection before TTV assessment	1.11	1.06‐1.15
	Tacrolimus trough level at TTV assessment	1.11	1.06‐1.15
	Full dose mycophenolic acid at TTV assessment	1.11	1.06‐1.15
	Prednisolone dose at TTV assessment	1.11	1.07‐1.16
	Estimated glomerular filtration rate a at TTV assessment	1.11	1.06‐1.15
	Leukocyte count at TTV assessment	1.10	1.06‐1.15
Final model	Recipient age and sex, CMV prophylaxis, ABO incompatible transplantation, preformed DSA, posttransplant diabetes mellitus, graft rejection before TTV assessment, leukocyte count, tacrolimus trough level, full dose mycophenolic acid at TTV assessment, time between kidney transplantation and TTV assessment	1.09	1.04‐1.14

Abbreviations: CMV, cytomegalovirus; DSA, donor‐specific antibodies; TTV, torque teno virus.

Calculated by the Modification of Diet in Renal Disease equation.^38^

## 4 DISCUSSION

This large prospective trial is the first report demonstrating the value of TTV for risk prediction of both, graft rejection and infection after month 3 posttransplant. TTV has been quantified weeks before rejections were diagnosed by biopsy or infections were clinically overt. Moreover, the study was able to define cutoff levels for an optimal TTV range and thus provides basis for a randomized controlled interventional trial assessing the efficacy of TTV‐guided immunosuppression to reduce graft rejection and infection.

A previous prospective analysis on TTV and infection in kidney transplant recipients was reported by Fernández‐Ruiz and colleagues.^25^ Due to a 3‐fold amount of available TTV measurements and infectious events we were able to extend the analysis to all clinically relevant infections and performed detailed subgroup analysis: the present report was able not only to demonstrate an association between TTV and subsequent opportunistic infections, CMV, and polyomavirus but also extracellular bacterial infections and infections that did not require hospitalization. Two other prospective trials analyzed the value of TTV for risk prediction of graft rejection in kidney transplant recipients. The report by Solis and colleagues did not report Banff categories and it is not clear whether BL lesions have been included.^27^ The report by Fernández‐Ruiz did not specify how rejection was scored and included cases without biopsy but clinical response to corticosteroids.^25^ Our report was restricted to biopsy proven graft rejection with detailed information on Banff categories. Moreover, we performed a subgroup analysis excluding BL lesions and confirmed the association of TTV and graft rejection in a cohort restricted to ABMR and Banff TCMR I or higher. Of note, both previous reports on rejection^25^, ^27^ were restricted to TTV assessed pretransplant and at posttransplant month 1, whereas our report presents data from TTV quantified after month 3 posttransplant. Due to the large sample size and extensive documentation of clinical follow‐up data, we were able to exclude possible confounders on the effect size of the association between TTV and graft rejection and infection, respectively. Moreover, we demonstrated that the effect size of the association between TTV and infections was similar across sex and age groups.

A main goal of the present trial was to define the optimal TTV range for reduced risk of graft rejection and infection in the first year after kidney transplantation providing a basis for an interventional randomized controlled trial to evaluate the efficacy of TTV‐guided immunosuppression. For this purpose, we included TTV levels after month 3 posttransplant, because steady state does not occur before this time point. Patients with subsequent allograft rejection had a median TTV load of 3.5 × 10^6^ c/mL and a TTV load < 1.5 × 10^6^ c/mL corresponded to a specificity of 89% to detect rejection. In line with these findings, the same specificity of 89% was calculated for a cutoff of 1.8 × 10^6^ c/mL for the detection of rejection using data obtained from our previously published case control study.^24^ For TTV levels above 10^7^ c/mL, high NPVs were calculated and PPV was high below 10^5^ c/mL. Taken together, these data suggest an optimal lower TTV cutoff of 1 × 10^6^ c/mL to guide immunosuppressive therapy. In the present analysis, patients with subsequent infection had a median TTV load of 3.9 × 10^8^ c/mL, whereas patients without infection had a median of 2.6 × 10^7^ c/mL. TTV levels > 5.8 × 10^9^ c/mL corresponded to a specificity of 90% to detect infection. For TTV level up to 10^9^ c/mL, high NPVs were calculated for the detection of infection and the highest PPV was calculated above 10^10^ c/mL. These findings are in line with an analysis in lung transplant recipients^17^: The authors calculated a specificity of 91% for TTV level > 2 × 10^9^ c/mL to detect infection. In their report, TTV was also quantified at the Center for Virology at the Medical University of Vienna and thus absolute TTV values are well comparable between their study and our current trial. Taken together, our data suggest an upper TTV limit of 10^8^ c/mL as an optimal target for an interventional trial optimizing immunosuppression and defines high risk for infections at TTV level above 10^9^ c/mL. Fernández‐Ruiz and colleagues described a median TTV load of 6.3 × 10^6^ c/mL for patients at month 6 post–kidney transplantation with infectious or oncologic disease. In this respect it is important to note that the authors used a different PCR assay for TTV quantification (TTV R‐gene® kit; ARGENE®, bioMérieux). In general, TTV loads quantified by the TTV R‐gene® kit are lower compared to the in‐house assay applied in our study. Moreover, plasma samples collected during the present study were processed at the day of blood draw, whereas Fernández‐Ruiz and colleagues analyzed samples stored at −80°C. In line with our data, they described a similar delta in TTV level between patients experiencing infection and patients without infection. This aspect underscores the importance of standardized TTV quantification to enable comparability between laboratories.

The major strengths of the present trial are the prospective design, the large sample size, and the unselected cohort of consecutive kidney transplant recipients. Detailed and prospective documentation of clinical characteristics allowed for the analysis of possible confounders and effect modifiers. The major limitation of the study is the single center design. Second, follow‐up was limited to 12 months after transplantation, and third, the number of biopsy proven rejections was small. Finally, the present data suggest independent association of TTV and rejection and infection due to level of immunosuppression, respectively, but did not prove causality. Similar to reports by Spanish^25^ and French groups^27^ diagnostic accuracy of TTV load in our trial does not allow for accurate diagnosis of subsequent infection or rejection but rather defines patients at risk. Therefore, TTV is not able to serve as a diagnostic parameter for infection and graft rejection but represents a promising candidate for personalization of immunosuppression.

Taken together the results of our study provide evidence for the value of TTV quantification for risk stratification of clinically relevant graft rejection and infection after kidney transplantation. Moreover, we defined an optimal TTV range as a basis for an interventional trial to test the efficacy of TTV‐guided immunosuppression reducing infection and rejection after kidney transplantation.

## DISCLOSURE

The authors of this manuscript have no conflicts of interest to disclose as described by the *American Journal of Transplantation*.

## Data Availability

The data that support the findings of this study are available from the corresponding author upon reasonable request.
