# Supplementary material for: Torque teno virus for risk stratification of graft rejection and infection in kidney transplant recipients—A prospective observational trial
Source: Am J Transplant. 2020 Mar 8;20(8):2081–90. doi: 10.1111/ajt.15810 (PMC7496119; doi:10.1111/ajt.15810)
Supplement: Supplementary file 1 [file AJT-20-2081-s001.pdf]

SUPPLEMENTARY FIGURE 1

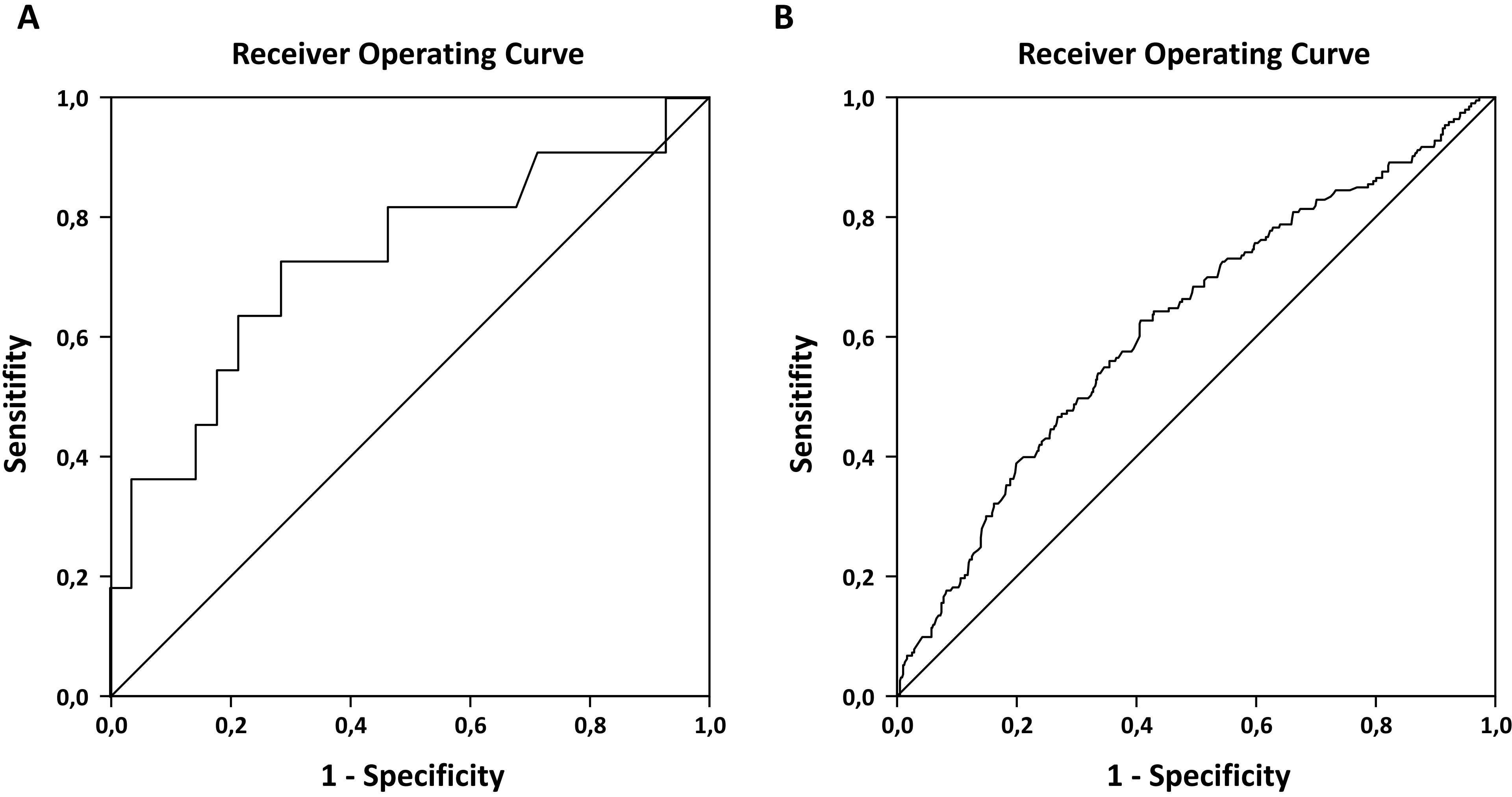

SUPPLEMENTARY FIGURE 1A Receiver operating curve was applied to classify graft rejection (A) and infection (B) by peripheral TTV load, respectively. Sensitivity is plotted on the y-axis and 1-specificity is plotted on the x-axis.
