# Supplementary material for: Torque teno virus for risk stratification of graft rejection and infection in kidney transplant recipients—A prospective observational trial
Source: Am J Transplant. 2020 Mar 8;20(8):2081–90. doi: 10.1111/ajt.15810 (PMC7496119; doi:10.1111/ajt.15810)
Supplement: Supplementary file 2 [file AJT-20-2081-s002.docx]

**SUPPLEMENTARY TABLE 1.** Cause of graft loss and death in the total cohort in year one after transplantation.

|  | **Number** |
| --- | --- |
| Cause of death | 17 |
| Cardiovascular disease | 7 |
| Infectious disease | 6 |
| Malignant disease | 3 |
| Unknown | 1 |
| Cause of graft-loss | 20 |
| Surgical complication | 9 |
| Pre-renal (hypo-perfusion) | 3 |
| Venous thrombosis | 2 |
| Donor malignoma | 1 |
| Infection | 1 |
| cABMR, collapsing glomerulopathy | 1 |
| ABMR with TMA | 1 |
| Infarction of unknown cause | 1 |
| Arterial occlusion | 1 |

Abbreviations: ABMR, antibody-mediated rejection; cABMR, chronic-active antibody-mediated rejection; TMA, thrombotic microangiopathy.

**SUPPLEMENTARY TABLE 2.** Rejection episodes classified according to BANFF in the total cohort and the cohort selected to analyze the association between TTV and rejection.

| **BANFF Category** | **Total cohort**  **(n = 71)** | | | | | **Biopsy cohort**  **(n = 11)** | | |  |
| --- | --- | --- | --- | --- | --- | --- | --- | --- | --- |
|  |  | |  | |  | |  | |  |
| T-cellular mediated rejection | 46 | (65) | | 5 | | | | (45) | |
| BL | 34 |  | | 2 | | | |  | |
| TCMR I | 6 |  | | 3 | | | |  | |
| TCMR II | 4 |  | | 0 | | | |  | |
| TCMR III | 0 |  | | 0 | | | |  | |
| cTCMR | 2 |  | | 0 | | | |  | |
| Antibody mediated rejection | 19 | (27) | | 2 | | | | (18) | |
| ABMR | 18 |  | | 2 | | | |  | |
| cABMR | 1 |  | | 0 | | | |  | |
| Mixed rejection | 8 | (11) | | 4 | | | | (36) | |
| ABMR, BL | 1 |  | | 3 | | | |  | |
| ABMR, TCMR I | 1 |  | | 1 | | | |  | |
| ABMR TCMR II | 4 |  | | 0 | | | |  | |
| cABMR, BL | 1 |  | | 0 | | | |  | |
| cABMR, cTCMR | 1 |  | | 0 | | | |  | |

Data are presented as number (%).

Abbreviations: ABMR, antibody-mediated rejection; cABMR, chronic-active antibody-mediated rejection; BL, borderline rejection; TCMR, T-cellular-mediated rejection; cTCMR, chronic T-cellular-mediated rejection.
